# A fast method for extracting essential and synthetic lethality genes in GEM models

**DOI:** 10.1093/bioadv/vbaf127

**Published:** 2025-06-06

**Authors:** Francisco Guil, José M García

**Affiliations:** Parallel Computer Architecture Group, University of Murcia, CEIR Campus Mare Nostrum, Murcia 30100, Spain; Parallel Computer Architecture Group, University of Murcia, CEIR Campus Mare Nostrum, Murcia 30100, Spain

## Abstract

**Summary:**

Exploring and categorizing essential and synthetic lethality genes is crucial in developing effective and targeted therapies for various diseases. This endeavor hinges upon genetic minimal cut sets, which also find utility in metabolic engineering. Different methods have been suggested for calculating genetic minimal cut sets. Still, with the emergence of numerous new models and their increasing complexity, it has become essential to introduce new algorithms in this field. This paper presents a new algorithmic approach for computing genetic minimal cut sets, which utilizes linear programming techniques to improve temporal efficiency. The key concept of the method is to use a k-representative subset to replace the target set with a smaller, yet representative, one. We have analyzed its efficiency in terms of running times compared to gMCSPy, the most recent published research on computing genetic minimal cut sets.

**Availability and implementation:**

Software and additional material are freely available at https://github.com/biogacop/fastMethod

## 1 Introduction

The remarkable progress in DNA sequencing has paved the way for genome-scale models, constituting a significant breakthrough in genetics that opens up new avenues for research.

Constraint-based modeling has been developed as a generalized approach to generate and study these models. One of the key concepts in this approach is that of minimal cut sets (MCS) (Klamt 2006, Hädicke and Klamt 2011). An MCS is a (minimal) set of reactions that, when inhibited simultaneously, prevent a specific task from being performed. It has been used to support the targeted design of microbial strains for bio-based production (Harder *et al.* 2016, von Kamp and Klamt, 2017, Alter and Ebert, 2019, Banerjee *et al.* 2020), to prevent the proliferation of certain bacteria (Guil *et al.* 2022) or for the discovery of potential targets for cancer (Tobalina *et al.* 2016, Apaolaza *et al.* 2017). Implementing deletion strategies at the genetic level is often challenging due to conflicts that arise when considering gene-protein rules (GPRs) within a network. As a result, the concept of MCS has expanded to include a minimal genetic cut set (gMCS). A gMCS is a minimal set of genes that, when inhibited, prevent certain states or modes of the network (Machado *et al.* 2016, Apaolaza *et al.* 2017, Schneider *et al.* 2020). This concept of gMCS is also referred to as essential or synthetic lethal genes in the biomedical field, where there is one or more genes, respectively.

In recent years, several methods have been developed to compute gMCSs (refer to section genetic minimal cut sets [gMCS]). However, most of these methods employ mixed integer linear programming (MILP) approaches for analysis, which can be time-consuming and resource-intensive and may lack numerical stability. However, due to the growing intricacy and quantity of models accessible (Robinson *et al.* 2020, Chen *et al.* 2022), introducing fresh algorithms has become a crucial objective in this area. New methods must efficiently manage large networks and implicit targets and execute quickly. This is especially vital in medical contexts, where utilizing multiple interconnected models to analyze differences between normal and pathological cases is essential, as mentioned in Foguet *et al.* (2022), Gustafsson *et al.* (2023).

Our paper introduces a new technique for calculating genetic MCSs. We achieve this by limiting the search space and computing hitting sets for a specific subset of the target set. Our approach has proven highly effective for identifying gMCSs with fewer genes (typically up to 4). In most cases, we have achieved efficiency rates that surpass those of previous methods.

The key concept is that of a k-representative subset of a target set **T** for a particular integer k≥1. A subset **T′** of **T** is said to be k-representative if it has precisely the same gMCSs of cardinality ≤k as **T**, so we can compute those gMCSs in **T′** instead of using the whole set **T**. We have created an algorithm that builds k-representative subsets iteratively. The process starts by computing a 1-representative subset and then successively extending it to k-representative subsets as k increases. The extensions are obtained by modifying the Berge algorithm (Berge 1984) and filtering the resulting hitting sets using linear optimization problems (LP). Substituting MILP problems with linear programming (LP) ones accelerates the method compared to previously proposed techniques.

As primary case studies, we use this new method to calculate synthetic lethality of length ≤4 in two different networks: *iML1515*, a reconstruction model for *Escherichia coli* (Monk *et al.* 2017), and *Human1*, a unified human GEM lineage (Robinson *et al.* 2020). We also calculate genetic interventions that ensure the coupled growth of biomass and ethanol in anaerobic conditions for the model *iJO1366*, another reconstruction model for *Escherichia coli* (Orth *et al.* 2011).

The *iML1515* and *iJO1366* models are available from BIGGs [Schellenberger *et al.* 2010, (https://bigg.ucsd.edu/)] while the Human1 model can be obtained from Metabolic Atlas [Li *et al.* 2023, (https://github.com/SysBioChalmers/Human-GEM)].

## 2 Material and methods

### 2.1 Metabolic networks

A metabolic network is represented by a tuple comprising three elements: *M*, *R*, and *S*. *M* and *R* are sets that represent the metabolites and reactions, respectively. Meanwhile, *S* is a stoichiometry matrix that belongs to $M_{m\times n}(R)$. This matrix serves as a link between the metabolites and reactions in the network. The values of *m* and *n* represent the number of internal metabolites and reactions, respectively. Each state of the network is represented by a flux vector $v\in R^{n}$, where $v_{i}$ represents the activity level of reaction $r_{i}$.

Let x represent the vector of metabolite concentrations; the variations in these concentrations are summarized in Equation (1)
 

(1) $$\frac{dx}{dt}=S\cdot v$$

The steady-state constraint’s equation is (2), where internal metabolite concentrations remain constant over time.

(2) S·v=0

Only internal metabolites must be included as rows in this formulation’s stoichiometric matrix *S*.

Each reaction in R has upper ($u_{i}$) and lower ($l_{i}$) bounds on its reaction rates $v_{i}$. Therefore, Equation (3) constrains any flux vector.

(3) $$l_{i}\leq v_{i}\leq u_{i};\;\forall r_{i}\in R$$

A vector is called feasible or a network mode if it satisfies Equations (2) and (3). The collection of all modes of the network is referred to as its feasible cone and is denoted by

$$E=\{v\in R^{n}|\;S\cdot v=0,\;l_{i}\leq v_{i}\leq u_{i},\;\forall r_{i}\in R\}$$

Given a mode v∈E, its support is the set of reactions that appear with nonzero flux in *v*:

$$\mathrm{supp}(v)=\{r_{i}\in R\;|\;v_{i}\neq 0\}$$

### 2.2 Genetic minimal cut sets

To begin, select a set of undesired modes, denoted as T and called a target set. A cut set for T, called C, is a combination of reactions that addresses all the undesired modes. In other words, C must intersect with every element v∈T. A MCS C is defined as one where no proper subset of C serves as a cut set for T (Hädicke and Klamt, 2011).

Metabolic networks often contain gene information through GPRs. GPRs for a reaction, denoted as *r*, are Boolean expressions indicating which gene combinations must be active to allow flux through *r*.

These GPRs extend the concept of MCSs to gMCS (Machado *et al.* 2016, Apaolaza *et al.* 2017).

A genetic cut set (gCS) for a target set **T** is a set of genes *G* that, when knocked out, renders none of the elements v∈  **T** a valid mode for the modified network. A gCS for **T** is minimal, i.e. gMCS, if it does not contain any proper subset that is also a gCS for **T**.

### 2.3 Using gMCSs to tackle various genetic strategies

Usually the set **T** is not explicitly given by a list of supports, but rather by inequalities set forth by a matrix $A\in R^{s\times n}$ and a vector $b\in R^{s}$ such that the set can be defined as **T**={v∈E | A·v≥b} (Schneider *et al.* 2020).

For this target set and a given set of genes, *G*, it is straightforward to determine if *G* is a gCS for **T**.

PREPOSITION 1. *Let*  ***T***={v∈E | A·v≥b}*, where*  $A\in R^{s\times n}$  *and*  $b\in R^{s}$*. Take G, a set of genes in the model. If we block all genes in G from the model and select any linear function f on the reactions, then G is a gCS for*  ***T***  *if and only if the following optimization problem is infeasible*
 

(4) $$\begin{array}{ccc} \text{Maximize} & f & \\ \text{subject}\;\text{to} & S\cdot v=0 & \\ & l_{i}\leq v_{i}\leq u_{i} & \forall r_{i}\in R\\ & A\cdot v\geq b & \end{array}$$

This paper applies this result to two distinct target sets: all modes with positive growth and without coupling between growth and a specific bioproduct production.

Let’s begin by discussing how to block all modes with positive growth. We focus on modes with nonzero flow through the biomass reaction, denoted as $r_{\text{biomass}}$. As this region is not defined by a restriction of the form A·v≥b, we will first calculate the maximum flow through $r_{\text{biomass}}$, resulting in a value of $r_{\text{biomass}}^{\text{max}}$. We define the set **T** as the set of modes whose biomass values exceed $p\cdot r_{\text{biomass}}^{\text{max}}$, where *p* is a proportion that we will set at p=0.01.

$$T=\{v\in E\;|\;r_{\text{biomass}}\geq p\cdot r_{\text{biomass}}^{\text{max}}\}$$

On the other hand, if we aim to promote the coupled growth of biomass and a byproduct linked to an exchange reaction *r*, our goal is to remove any mode enabling flux through $r_{\text{biomass}}$ without carrying flux through *r*. Again, we will determine the maximum flux through $r_{\text{biomass}}^{\text{max}}$ and use as target set

$$T=\{v\in E\;|\;v_{\text{biomass}}\geq p\cdot r_{\text{biomass}}^{\text{max}},v_{r}=0\}$$

Where *p* represents the same proportion constant as in the previous scenario.

It’s important to note that any genetic intervention targeting this set **T** falls into one of two categories:

- Interventions blocking the biomass reaction.
- Interventions that ensure the bioproduct production whenever $r_{\text{biomass}}>p\cdot r_{\text{biomass}}^{\text{max}}$.

We focus only on interventions in the second class, but it is easier to calculate all interventions and filter them by their maximum biomass flux.

### 2.4 Computing gMCSs

Multiple methods have been suggested for calculating MCSs and gMCSs. A straightforward method is to test all increasing-length gene combinations to detect and filter the minimal gCS by inclusion. This approach is limited to small cardinalities, typically no more than two genes, but can be enhanced by narrowing the search space. This field of study originated with the SL-Finder algorithm (Suthers *et al.* 2009) and has since been applied to other algorithms, such as fastSL (Pratapa *et al.* 2015) and rapidSL (Dehghan *et al.* 2022).

A second approach relies on computing (a base of) the target set and computing MCSs as hitting sets of their supports, often using a variation of Berge’s algorithm (Berge, 1984, Jungreuthmayer *et al.* 2013a, b). These methods typically rely on a large base set, making them impractical for use in large networks.

New methods were developed to utilize the relationship between cut sets of the network and elementary flux modes of a specific dual network. This approach was initially suggested in Ballerstein *et al.* (2012). This method can also be applied to gMCSs by adding genes to the network, as shown in Machado *et al.* (2016) and Schneider *et al.* (2020). Alternatively, a gene matrix can be introduced, as described in Apaolaza *et al.* (2017) and Apaolaza *et al.* (2019). This gene matrix is a binary matrix G that defines for each row the set of blocked reactions arising from the knockout of a particular subset of genes. This line of research has led to the development of gMCSPy, the latest algorithm for computing gMCSs (Rodriguez-Flores *et al.* 2024). These methods rely on solving MILP, resulting in high time and resource costs.

We have developed a new method for computing gMCSs through a multi-step process. First, we compute gMCSs for specific reactions. Next, we extend this approach to include modes and sets of modes. Finally, we utilize k-representative subsets of the target set to calculate the gMCSs for target sets of the form efficiently **T**={v∈E | A·v≥b} for certain $A\in R^{s\times n}$ and $b\in R^{s}$.

#### 2.4.1 Computing gMCSs for reactions and modes

We begin our approach by examining the process of identifying gCSs and gMCSs for individual reactions.

Usually, the GPR for a given reaction r∈R comes in two forms: a sum of products disjunctive normal form (DNF) or a product of sums, conjunctive normal form (CNF).

- Notice that if the GPR is in DNF form, any GPR can be easily converted to CNF form for the sets of genes corresponding to the summands.
- If the GPR for *r* is in DNF form, then its gCSs are the supersets of the sets of genes corresponding to each factor.

For our purposes, the second type of expression is more desirable. Moreover, the following algorithm makes converting GPRs from DNF to CNF form easy.

- Begin with a reaction *r* that has a GPR in DNF form.
- Express each term as a collection of genes.
- The GPR for *r* can be expressed as the product of sums of minimal hitting sets. These sets can be computed using Berge’s algorithm.

After being transformed into CNF form, the gMCSs for *r* are the factors of its GPR that do not contain any other factor as a subset.

It is important to note that if we want to focus on gCSs with length ≤k for some natural number 1≤k∈N, we can limit ourselves to factors of these GPRs containing at most *k* terms.

Example 1. *Consider the two following examples:*
 

1. *Define the metabolic model containing 5 metabolites, 8 reactions, and 9 genes given in the following figure*
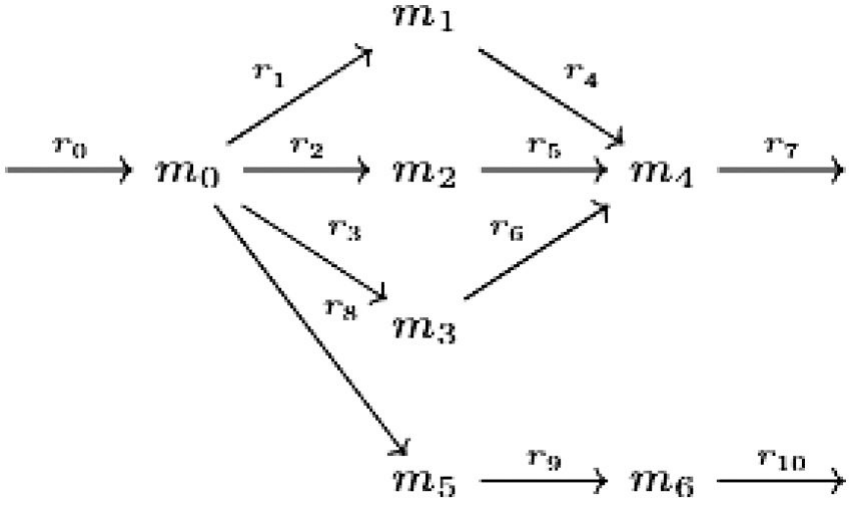
  Where the GPRs for these reactions can be found in *Table* 1.
   *The GPR for reaction*  $r_{7}$  *is in CNF. Its gMCSs are the factors*  $\left\{g_{0}\right\}$  *and*  $\left\{g_{5},g_{6},g_{7}\right\}$.
   *However, the GPR for*  $r_{9}$  *is expressed in disjunctive normal form (DNF). According to the algorithm described above, it can equivalently be stated as*  $\left(g_{1}\vee g_{3})\wedge (g_{1}\vee g_{4})\wedge (g_{2}\vee g_{3})\wedge (g_{2}\vee g_{4}\right)$*, so it has four gMCSs corresponding to its factors.*
2. *The model* Human1 *version 1.16 contains 13085 reactions, of which 8091 have an associated GPR rule. Of these, 8087 rules are in DNF form, while only 4 are in CNF form.*
   *The GPR of reaction MAR07161 is in CNF form and includes twenty-eight factors with length 1, one factor with length 2, one with length 3, and one with length 4. As a result, there are thirty-one gMCSs for this reaction with lengths 1, 2, 3, and 4, each corresponding to the different factors.*
   *The GPR for reaction MAR04611 is in DNF form and consists of two summands, each with four genes. Three genes are present in both summands. After being converted to CNF, it contains four factors corresponding to its gMCSs. These gMCSs consist of three gMCSs of length 3 and one gMCS of length 2.*

**Table 1.** The gene-protein rule for all reactions in the model is presented in CNF form.

Reaction	GPR	Reaction	GPR
$r_{0}$	$g_{0}$	$r_{5}$	$g_{4}$
$r_{1}$	$g_{1}$	$r_{6}$	$g_{4}$
$r_{2}$	$g_{1}$	$r_{7}$	$g_{0}\;\wedge \;(g_{5}\;\vee \;g_{6}\;\vee \;g_{7})$
$r_{3}$	$g_{2}$	$r_{8}$	$g_{8}$
$r_{4}$	$g_{3}$	$r_{9}$	$\left(g_{1}\;\wedge \;g_{2})\;\vee \;(g_{3}\;\wedge \;g_{4}\right)$

We will use gCS(v), $gCS^{k}(v)$, gMCS(v), and $\mathrm{gMC}S^{k}(v)$ to denote the sets of gCSs, gCSs of length ≤k, gMCSs and gMCSs of length ≤k for a given mode v∈E, respectively. We extend the same notations for a target set of modes **T**.

To expand the computation of gCSs from reactions to modes, it should be noted that a set of genes, *G*, is a gCS for a given mode *v* if and only if there exists a reaction *r* within the support of *v* such that *G* is a gCS for *r*. So, we have

$$\mathrm{gCS}(v)=\underset{r\in \mathrm{supp}(v)}{\cup }gCS(r)$$

and

$$gCS^{k}(v)=\underset{r\in \mathrm{supp}(v)}{\cup }gCS^{k}(r)$$

We introduce the concept of a reduced set of gene sets to characterize gMCSs for a given mode *e*.

Definition 1. A set of gene sets, *K*, is reduced if each set G∈C has no proper subset $G^{\prime }\in C$.

 Given a set of gene sets *K*, its reduction is defined as $\bar{K}=\{G\in K\;|\;G\;\text{is}\;\text{minimal}\}$

 The gMCSs for a specific mode *v* are characterized by Preposition 2.

PREPOSITION 2. *Let*  v∈E  *be a mode of the network. Then*
 

- gMCS(v)  *is the reduction of*
 
  $$\underset{r\in \mathrm{supp}(v)}{\cup }gMCS(r)$$
- *For any*  1≤k∈N, $\mathrm{gMC}S^{k}(v)$  *is the reduction of*
 
  $$\underset{r\in \mathrm{supp}(v)}{\cup }gMCS^{k}(r)$$

#### 2.4.2 Calculating gMCSs for sets of modes

We can now compute gCSs for a target set of modes **T**.

PREPOSITION 3. *The set of gCSs for a target set of modes*  ***T***  *is the intersection of the gCSs of each mode in*  ***T***.

$$\mathrm{gCS}(T)=\underset{v\in T}{\cap }gCS(v)$$

However, this statement no longer applies to gMCSs because a gMCS of a mode v∈T can contain a gMCS of another mode $v^{\prime }\in T$ as a subset.

To clarify this question, let’s rephrase our definition of a gCS for the target set **T**. A set of genes *G* is a gCS for **T** if it is a gCS for any element *e* in **T**. In other words, for each mode *v* in **T**, there exists at least one element $G^{\prime }$ in gMCS(v) such that $G^{\prime }\subset G$.

This description can be viewed as an extension of the idea of a hitting set.

Definition 2. We say that *G* is a hitting set for **T** if, for any v∈  **T**, there exists an element $G^{\prime }\in \mathrm{gMCS}(v)$ such that $G^{\prime }$ is a subset of *G.*

 So, GCSs refer to the hitting sets for T. At the same time, gMCSs can be recognized as the minimal hitting sets for T. Identifying gMCSs with minimal hitting sets has the advantage of being easily adaptable to the Berge algorithm for their detection (refer to Algorithm 1).

Algorithm 1. Modified Berge algorithm

**Require:** A set of modes **T**

**Ensure:** The set of gMCSs for **T**

 Initialization gMCS={∅};

 **for**  v∈  **T do**

   **for**  G∈gCS  **do**

    Check if *G* is a superset of some $G^{\prime }\in \mathrm{gMCS}(v)$  

     **if** False **then**

      **for**  $G^{\prime }\in \mathrm{gMCS}(v)$  **do**

      $\mathrm{gMCS}\leftarrow G\cup G^{\prime };$

      **end for**

      Remove *G* from gMCS

     **end if**

  **end for**

  **for** *G* in gMCS **do**

    **if** There exists $G^{\prime }$ in gMCS with $G^{\prime }\subset G$  **then**

       Remove *G* from gMCS

    **end if**

  **end for**

**end for**

Example 2. *Let’s continue with Example 1. Consider the set of modes denoted by*  ***T***  *as follows:*
 

$$\left\{\{r_{0},r_{1},r_{4},r_{7}\},\{r_{0},r_{2},r_{5},r_{7}\},\{r_{0},r_{3},r_{6},r_{7}\}\}\subset \{e\in E\;|\;v_{10}=0\right\}$$

*For all reactions except*  $r_{7}$*, the genetic support consists only of its associated gene. For reaction*  $r_{7}$*, its genetic support is*
 

$$\mathrm{gMCS}(r_{7})=\{\{g_{0}\},\{g_{5},g_{6},g_{7}\}\}$$

*For any mode in*  ***T***  *we have:*
 

- $\mathrm{gMCS}(\{r_{0},r_{1},r_{4},r_{7}\})=\{\{g_{0}\},\{g_{1}\},\{g_{3}\},\{g_{5},g_{6},g_{7}\}\}$
- $\mathrm{gMCS}(\{r_{0},r_{2},r_{5},r_{7}\})=\{\{g_{0}\},\{g_{1}\},\{g_{4}\},\{g_{5},g_{6},g_{7}\}\}$
- $\mathrm{gMCS}(\{r_{0},r_{3},r_{6},r_{7}\})=\{\{g_{0}\},\{g_{1},g_{2}\},\{g_{3},g_{4}\},\{g_{5},g_{6},g_{7}\}\}$

*It is easy to use Algorithm 1 to check that*  ***T***  *has exactly five gMCSs:*  $\left\{\{g_{0}\},\{g_{1},g_{2}\},\{g_{1},g_{4}\},\{g_{3},g_{4}\},\{g_{5},g_{6},g_{7}\}\right\}$.

### 2.5 Computing gMCSs for large target sets

Consider a target set given by **T**={v∈E | A·v≤b} for certain $A\in R^{s\times n}$ and $b\in R^{s}$. If we want to use Algorithm 1 to compute its gMCSs, the primary challenge in this process is to calculate all the modes (or EFMs) present in **T**, which is a time-consuming task for most networks (if possible).

#### 2.5.1 k-representative subsets

We recommend computing a smaller subset of modes called **T′** from the more extensive set **T** to solve this issue. By doing this, we can use the gMCSs of **T′** as an in-between step to identify the gMCSs for **T**.

To begin, we need to analyze the relationship between the gMCSs of a target set **T** and a subset **T′**⊂**T**.

PREPOSITION 4. *The following properties apply to any*  $T^{\prime }\subset T$*:*
 

- *Any gCS for*  ***T***  *is also a gCS for*  ***T′***
- *Any gCS for*  ***T***  *must contain a gMCS for*  ***T′***
- *If a gMCS for*  ***T′***  *is a gCS for*  ***T***  *then it is also a gMCS for*  ***T***

According to Proposition 4, we can calculate the gMCSs for **T** by first computing the gMCSs for **T′** and eliminating those that are not gMCSs for **T**. To guarantee that the resulting gCS are minimal, we should compute them in increasing length. Thus, we can verify that every computed gCS includes no other gMCS with a smaller length.

However, there is a potential obstacle that we need to avoid. Not all gMCSs for **T′** of a given length are also gMCSs for **T**. So, if we are computing gCSs for T′ to use as potential gMCSs for **T** of length *k*, we cannot eliminate those that contain any gMCS for **T′** of length ≤k−1. We need to identify the gMCSs for T and exclude any gCSs for T′ that contain them. If $G^{\prime }$ is a gMCS for **T′** whose length is less than *k*, then any superset *G* of $G^{\prime }$ will also be **T′**. This set may not be filtered out, so we must consider it a potential candidate for being a gMCS for **T**. As a result, we will receive many candidates to be gMCSs for **T**.

Example 3. *Following with Example 1, consider*
 

$$T^{\prime }=\{\{r_{0},r_{1},r_{4},r_{7}\}\}\subset T$$

*Suppose we are interested in computing those gMCSs of length*  ≤2  *for*  ***T***.

 *We compute the gMCSs of length 1 for*  ***T′***  *as*  $\left\{\{g_{0}\},\{g_{1}\},\{g_{4}\}\right\}$*. Only*  $\left\{\{g_{0}\}\right\}$  *is a gMCS for*  ***T***.

 *Let’s calculate the gCSs of length 2 for T′, which are:*
 

$$\begin{array}{c} \{\{\{g_{0},g_{1}\},\{g_{0},g_{2}\},\{g_{0},g_{3}\},\{g_{0},g_{4}\},\{g_{0},g_{5}\},\{g_{0},g_{6}\},\\ \{g_{0},g_{7}\},\{g_{1},g_{2}\},\{g_{1},g_{3}\},\{g_{1},g_{4}\},\{g_{1},g_{5}\},\{g_{1},g_{6}\},\\ \left\{g_{1},g_{7}\},\{g_{4},g_{2}\},\{g_{4},g_{3}\},\{g_{4},g_{5}\},\{g_{4},g_{6}\},\{g_{4},g_{7}\}\right\} \end{array}$$

*To simplify the gCS for*  ***T****, we can eliminate any gCS containing only the unique gMCS of length 1*, $g_{0}$*. However, we cannot eliminate gCSs that contain either*  $g_{1}$  *or*  $g_{2}$  *as they are not gMCSs for*  ***T****. For instance*, $\left\{g_{1},g_{2}\right\}$  *is a legitimate gMCS for*  ***T***  *even though it includes*  $g_{1}$.

It is essential to acknowledge that this is a significant concern. If we denote the number of genes in the network as *s*, for each $G\in \mathrm{gMCS}(T^{\prime })\backslash \mathrm{gCS}(T)$ of length k,’ we can obtain up to $\left(\begin{array}{c} s-1\\ k-k^{\prime } \end{array}\right)$ candidates that may belong in $\mathrm{gMC}S^{k}(T)$.

To solve this issue, we propose the idea of a k-representative subset **T′**⊂  **T**.

Definition 3. A set of modes **T′** is k-representative if any gMCS for **T′** of length ≤k is also a gMCS for **T**.

Example 4. *The subset T′ defined in Example 3 was not 1-representative because there were gMCSs of length 1 for T′ that are not gMCSs for T.*

 *On the other hand*, ***T″***  *=*  $\left\{\{r_{0},r_{3},r_{6},r_{7}\}\right\}$  *is 1-representative, because it has only one gMCS of length 1*, $\left\{g_{0}\right\}$*, which is also a gMCS for*  ***T***.

According to Definition 3, if a subset **T′**  ⊂  **T** is k-representative of the target region **T**, then a gMCS of length k+1 for **T′** is also a gMCS for **T** if it is a gCS for **T**.

#### 2.5.2 Computing gMCSs for T using k-representative subsets

We have all the necessary components to begin computing gMCSs for a target set **T**={v∈E | A·v≥b} for certain $A\in R^{s\times n}$ and $b\in R^{s}$.

We begin by finding a k-saturated subset T′ of T for a particular integer k, where k is a natural number. Choose any nonzero mode v∈T to accomplish this.

Please note that if gMCS(v)=∅, then according to 4, there are no gMCS for **T** as well. In all other scenarios, determine the smallest length of elements in gMCS(v) as *k* and identify all gMCSs with length *k*. While {v} may not be k-representative, it can be easily extended to a *k*-representative set **T′**. Start by creating $T^{\prime }=\{v\}$. For any $G_{i}\in \mathrm{gMC}S^{k}(v)$, knock out all genes in $G_{i}$ and choose a linear function *f* on the reactions. Then, pose the LP problem:

(5) $$\begin{array}{ccc} \text{Maximize} & f & \\ \text{subject}\;\text{to} & S\cdot v=0 & \\ & v_{i}\geq 0 & \forall r_{i}\in \mathrm{Irr}\\ & T\cdot v\geq t & \end{array}$$

If the problem cannot be solved, $G_{i}$ becomes a gMCS for **T**. If we can solve it, we find a mode $v^{i}\in$  **T** such that $G_{i}$ is not a cut set for $v^{i}$. We add the mode $v^{i}$ to **T′**.

Through this process, we have computed all the gMCSs of length k for T and a k-saturated subset T′.

PREPOSITION 5. ***T′***  *is a subset of*  ***T***  *that is k-saturated.*

PROOF.

It is worth noting that, according to Proposition 4, any set $G^{i}$ belonging to $\mathrm{gMC}S^{k}(T^{\prime })$ can also be found in $\mathrm{gMC}S^{k}(\{v\})$. If $G^{i}$ was not a gMCS for *T*, we know there is a mode $v^{i}$ such that $G^{i}$ is not a gCS for $v^{i}$. This contradicts the assumption that $G^{i}$ belongs to $\mathrm{gMCS}(T^{\prime })$. ▪

The steps to construct *k*, all $\mathrm{gMC}S^{k}(T)$, and the k-saturated subset $T^{\prime }\subset T$ are outlined in Algorithm 2.

Algorithm 2. Algorithm for computing all *k* gMCSs for **T** while also constructing a *k*-representative set

**Require:** A set of modes **T**

**Ensure:** A *k*-representative set of modes **T’** and $\mathrm{gMC}S^{k}(T)$

  Initialization: **T′**={v} for some v∈  **T**;

  k=min(length(G) | G∈gMCS(v));

  **for**  $G\in \underset{v\in T^{\prime }}{\cap }gMCS^{k}(v)$  **do**

     Check if *G* is a cut set for **T** 

     **if** False **then**

       Find $v^{\prime }\in$  **T** such that *G* is not a cut set for $v^{\prime }$ 

       **T′**←  **T′**$\cup \{v^{\prime }\}$  

     **else**

       *G* is a gMCS for **T** of length *k*

     **end if**

  **end for**

Suppose we have a subset **T′**⊂  **T** that is k-representative for i<k∈N and we have already computed all the gMCSs for **T** of length ≤k. We can expand the set T′ to a new set T″ such that T″ is a (*k* + 1)-representative subset of T while computing the gMCSs for T of length *k* + 1.

- Begin by setting $T^{\prime \prime }$ equal to $T^{\prime }$.
- Compute all gCSs of length k+1 for $T^{\prime }$ using Algorithm 1, and then filter them by removing any gCSs containing an element of $\mathrm{gMC}S^{k}(T)=\mathrm{gMC}S^{k}(T^{\prime })$. The remaining gCSs are candidates for $\mathrm{gMC}S^{k+1}(T)$.
- Proceed as in Algorithm 2 to check if candidate $G_{i}$ is in $\mathrm{gMC}S^{k+1}(T)$.
- In any other case, find a mode $v^{i}\in T$ such that $G_{i}$ is not a gCS for $v^{i}$. Add it to $T^{\prime \prime }$

The summarized process can be found in Algorithm 3.

Algorithm 3. algorithm to extend a k-representative set of modes to a (k + 1)-representative set

**Require:** A k-representative set of modes **T′** and $\mathrm{gMC}S^{k}(T)$

**Ensure:** A (k + 1)-representative set of modes **T″** and $\mathrm{gMC}S^{k+1}(T)$

   Initialization: T**″** = T′;

   Compute $\mathrm{gMC}S^{k+1}(T^{\prime \prime })$

   **for**  $G^{i}\in \mathrm{gMC}S^{k+1}(T^{\prime \prime })$  **do**

      Check if $G^{i}$ is a cut set for **T** 

      **if** False **then**

        Find $v^{i}\in$  **T** such that $G^{i}$ is not a cut set for $v^{i}$ 

        T**″**← T**″**$\cup \{v^{i}\}$ 

      **else**

        **if**  $G^{i}$ does not contain any gMCS of length

         ≤k  **then**

         $G^{i}$ is a gMCS for **T** of length k+1.

        **end if**

      **end if**

   **end for**

Combining both methods yields an algorithm that finds all gMCSs for **T** of length ≤k given an integer k≥1. Additionally, the algorithm computes a k-representative subset **T ′** of **T**.

Example 5. *Let’s utilize the model that was previously established in Example 2 using as target set*  $T=\{v\in E\;|\;v_{10}=0\}$

 *We obtain all gMCSs for T of length*  ≤3  *using Algorithms 2 and 3. We start by taking*  $v=\{r_{0},r_{1},r_{4},r_{7}\}\in T$, $T^{\prime }=\{v\}$.

 *Let’s compute*  gMCS(v)
 

$$\mathrm{gMCS}(v)=\{\{g_{0}\},\{g_{1}\},\{g_{3}\},\{g_{5},g_{6},g_{7}\}\}$$

 *Since*  k=1*, we can get*  $\mathrm{gMC}S^{1}(v)$  *which is equal to*  $\left\{\{g_{0}\},\{g_{1}\},\{g_{3}\}\right\}$.

 *We have three candidates for being gMCSs for*  ***T***  *of length*  k=1*. After solving the corresponding LP problems, we obtain the following:*
 

- $\left\{g_{0}\right\}$  *is a gMCS for*  ***T***.
- $\left\{g_{1}\right\}$  *is not a gMCS for*  ***T****. We obtain mode*  $v^{1}=\{r_{0},r_{2},r_{5},r_{7}\}\in$  ***T***  *with*  $\left\{g_{1}\right\}$  *not being a gCS for*  $v^{1}$*. We actualize*  ***T′***  *=*  $\left\{e,v^{1}\right\}$.
- $\left\{g_{3}\right\}$  *is not a gMCS for*  ***T′****. We obtain mode*  $v^{2}=\{r_{0},r_{3},r_{6},r_{7}\}\in$  ***T***  *with*  $\left\{g_{3}\right\}$  *not being a gCS for*  $v^{2}$*. We actualize*  ***T′***  *=*  $\left\{v,v^{1},v^{2}\right\}$.

 *So far, we have obtained a unique gMC of length 1*, $\left\{g_{0}\right\}$*, and the 1-representative subset*  ***T′****=*$\left\{v,v^{1},v^{2}\right\}$.

 *The next step is to calculate*  $\mathrm{gMCS}s^{2}(T^{\prime })$  *and expand*  ***T′***  *to a 2-representative subset of*  ***T***.

*We get*
 

$$\begin{array}{c} \sup p_{G}^{2}(v)=\{\{g_{0}\},\{g_{1}\},\{g_{3}\}\}\\ \sup p_{G}^{2}(v^{1})=\{\{g_{0}\},\{g_{2}\},\{g_{4}\}\} \end{array}$$

*Using Berge’s algorithm, the mGCS for T′ of length less than or equal to 2 are*  $\left\{\{g_{0}\},\{g_{1},g_{2}\},\{g_{1},g_{4}\},\{g_{2},g_{3}\},\{g_{3},g_{4}\}\right\}$*. We check if any of them are a gCS for T.*
 

- $\left\{\{g_{1},g_{2}\},\{g_{3},g_{4}\}\right\}$  *are gMCSs for*  ***T***
- $\left\{g_{2},g_{3}\right\}$  *is not a gMCS for*  ***T****. We get again the mode*  $v^{2}=\{r_{0},r_{3},r_{6},r_{7}\}\in T$  *such that*  $\left\{g_{3},g_{2}\right\}$  *is not a gMCS for*  $v^{2}$*. We do not need to update it as it is included in*  ***T′***.
- $\left\{g_{1},g_{4}\right\}$  *is not a gMCS for*  ***T′****. We get again the mode*  $v^{1}=\{r_{0},r_{3},r_{6},r_{7}\}\in T$  *such that*  $\left\{g_{2},g_{3}\right\}$  *is not a gMCS for*  $v^{1}$*. We do not need to update it as it is included in*  ***T′***.

 *We have completed the computation of*  $\mathrm{gMC}S^{2}(T)$*. Now*, ***T′***  *is a 2-representative subset of*  ***T***.

 *Next, we calculate the gMCSs with a length of 3. Beginning with*  $T^{\prime }=\{v,v^{1},v^{2}\}$*, we get:*
 

$$\begin{array}{c} \sup p_{G}^{3}(v)=\{\{g_{0}\},\{g_{1}\},\{g_{3}\},\{g_{5},g_{6},g_{7}\}\}\\ \sup p_{G}^{3}(v^{1})=\{\{g_{0}\},\{g_{2}\},\{g_{4}\},\{g_{5},g_{6},g_{7}\}\}\\ \sup p_{G}^{3}(v^{2})=\{\{g_{0}\},\{g_{1},g_{2}\},\{g_{3},g_{4}\},\{g_{5},g_{6},g_{7}\}\} \end{array}$$

 *By employing Berge’s algorithm once more, the gMCS for*  ***T*′**  *with length*  ≤3  *are*
 

$$\left\{\{g_{0}\},\{g_{1},g_{2}\},\{g_{1},g_{4}\},\{g_{5},g_{6},g_{7}\}\right\}$$

*They are all gMCS for*  ***T****. Therefore*,

$$\mathrm{gMC}S^{3}(T)=\{\{g_{0}\},\{g_{1},g_{2}\},\{g_{1},g_{4}\},\{g_{5},g_{6},g_{7}\}\}$$

## 3 Results

### 3.1 Test bed

The evaluation platform consisted of a double-socket Intel Xeon Gold 6226R processor with 32 physical cores (64 logical) and 384 GB of RAM, running on CentOS 8.2 (kernel version 4.18.0), provided by the Research Group of High-Performance Computer Architecture (GACOP) at the University of Murcia (Spain). All the results in the paper were carried out using the maximum number of cores, i.e. 64.

The network models were analyzed using the COBRApy package with Python 3.6 kernel in a Jupyter Notebook. We utilized Gurobi version 12.10 (https://www.gurobi.com) to solve the associated LP problems.

For comparison purposes, we have also shown the results obtained using the method described in Apaolaza *et al.* (2019) and based on a gene matrix. Specifically, we have used gMCSPy, the Python porting of this method (Rodriguez-Flores *et al.* 2024).

### 3.2 Network models used as case studies

We selected three network models as case studies to test our algorithm. We utilized two of them, namely *iML1515* and *Human1*, to compute their synthetic lethality. On the other hand, we used the third one to determine genetic interventions that can guarantee growth-coupling between biomass and ethanol.

The first reconstruction model used is *iML1515* for Escherichia coli, which has 2712 reactions, 1877 metabolites, and 1516 genes. This model can be found in Schellenberger *et al.* (2010) and was previously used in Schneider *et al.* (2020) to compare their algorithm’s efficiency in computing gMCSs to the one proposed in Apaolaza *et al.* (2017). Their study set the maximum length of the calculated gMCSs to *k* = 4. We also used this maximum length for all our cases.

The second model is the unified human GEM lineage, known as *Human1*. Its latest release (version 1.19) can be obtained from https://github.com/SysBioChalmers/Human-GEM and consists of 12971 reactions, 8455 metabolites, and 2887 genes.

Finally, we have explored genetic modifications to promote ethanol production from biomass in oxygen-free environments using the *iJO1366* model. This model encompasses 2583 reactions, 1805 metabolites, and 1367 genes.

### 3.3 Obtained metabolic interventions

We begin our study using the *iML1515* reconstruction model for Escherichia coli. This model contains 889 gMCSs that are of a length less than or equal to 4.

We calculated all gMCSs and recorded the runtimes while running the algorithm ten times using our method and gMCSPy to verify its correctness. Table 2 displays the number of gMCSs for each length and the meantime taken to compute all gMCSs up to that length in seconds.

**Table 2.** Time in seconds for obtaining synthetic lethality of length ≤4 in *iML1515* using our method and gMCSPy.

k	Number of gMCSs	FastMethod	gMCSPy
1	196	3,78	5,77
2	78	25,14	14,63
3	119	58,17	1 00,36
4	496	1 73,99	1612.13

**Figure 1.**
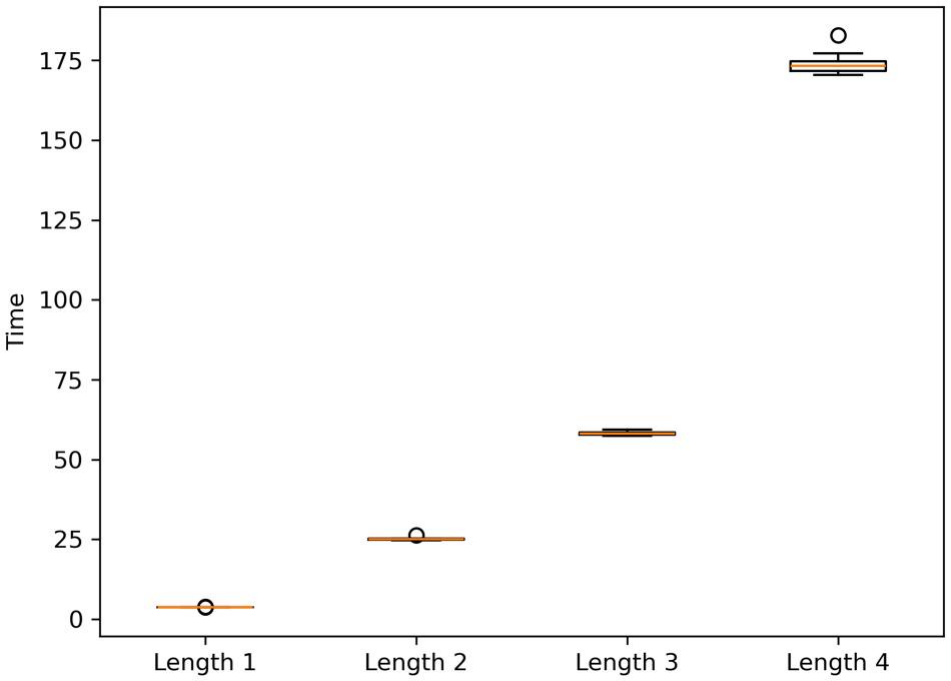
Boxplots illustrating the variation in execution times for computing all gMCSs of lengths ≤1, 2, 3, and 4 in *iML1515*.

Figure 1 illustrates the changes in execution times corresponding to each maximum length.

**Figure 2.**
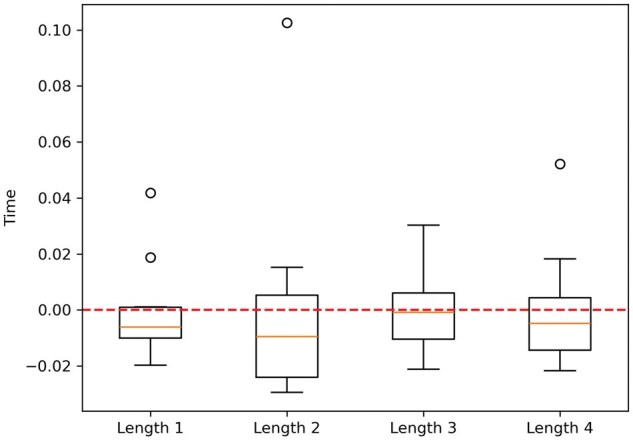
Boxplots showing the variation in the proportional execution times for calculating all gMCSs of lengths ≤1, 2, 3, and 4 in iML1515.

We can better understand the data by examining the variations from the mean. Specifically, we can substitute each execution time *t*, which was recorded while computing the gMCSs of length *l*, with the value $\frac{t-m}{m}$, where *m* represents the average execution time for that length *l*. Figure 2 shows this information. It can be observed that execution times are generally lower than the mean, with fluctuations not exceeding 3%.

We computed all gMCSs with length ≤4 for version 1.19 of the *Human1* model under Ham’s medium as a second example.

This model contains 2549 gMCSs with a length of four or fewer. Table 3 displays the number of gMCSs at each length. It also indicates the time in seconds needed to compute all gMCSs with lengths up to this value.

**Table 3.** Time in seconds for obtaining synthetic lethalities of length ≤4 in *Human1* under Ham’s medium using FastMethod and gMCSPy.

k	Number of gMCSs	FastMethod	gMCSPy
1	149	15,33	14,58
2	51	1 10,11	23,97
3	73	3 58,04	1 28,18
4	2276	8 43,09	11 00,63

**Figure 3.**
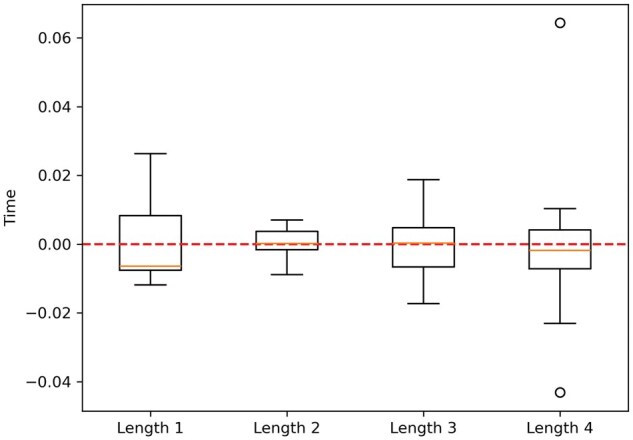
Boxplots showing the variation in the proportional execution times for calculating all gMCSs of lengths ≤1, 2, 3, and 4 in Human1.

Figure 3 illustrates the variations from the mean execution times recorded while calculating gMCSs of varying maximum lengths in *Human1* using Ham’s medium. It shows that the execution times are generally below the mean, with fluctuations remaining within 3%.

For our third example, we focused on exploring genetic interventions that could enable the coupling of ethanol and biomass growth under anaerobic conditions in the *iJO1366* model. First, we restricted the model’s ability to have flux through the exchange reaction linked to $O_{2}$ in this model.

We found 263 genetic interventions of length 4 or less. We ran the algorithm 10 times and recorded its run times in seconds. The corresponding results are shown in Table 4.

**Table 4.** Time in seconds for obtaining genetic interventions of length ≤4 in *iJO1366* for the couple growth of ethanol.

k	Interventions	Min time	Max time	Mean time
1	0	7,59	8,53	7,91
2	3	36.33	41,23	39,39
3	64	61,85	81,05	67,62
4	196	3 75,01	4 31,45	3 95,91

The Supplementary Material provides a more extensive comparison of the running times for our proposed method and gMCSPy across 28 different models.

## 4 Discussion

The gMCS framework is a powerful tool for exploring genomic-scale models.

When evaluating methods for computing gMCSs, it is essential to consider their ability to handle large networks and implicit target sets, while also obtaining reasonably short execution times. It would also be desirable to have a single technique that could address all related problems, including identifying synthetic lethal genes and suggesting genetic interventions for the growth coupling of byproducts.

Our algorithm has successfully met the first two criteria and has demonstrated effectiveness across various scenarios through multiple case studies. Regarding execution times, our method outperforms gMCSPy in 19 out of 28 models tested. Further details can be found in the Supplementary Material.

There are also instances where gMCSPy’s execution times outperform our algorithm, especially when the number of gMCSs is relatively low. The elapsed time depends on the model’s size, the number of gMCSs to be computed, their distribution, and the lengths of these gMCSs. Therefore, both methods have advantages and disadvantages and should be viewed as complementary.

## 5 Conclusions

A gMCS is a set of genes that, when inactivated, prevents any mode of a specific target set. The target set varies depending on the desired genetic intervention. This concept provides a comprehensive approach to examining essential and synthetic lethal genes, as well as metabolic engineering techniques, to ensure growth coupling for desired byproducts.

Numerous algorithms have been created to calculate gMCSs, with most relying on developing a dual network and utilizing MILP approaches for analysis. Regrettably, these techniques may lack numerical stability and can be costly in both time and resources.

In this paper, we present a novel algorithm for computing gMCSs. The new approach employs linear programming techniques, resulting in more efficient runtimes. The main idea is to use a k-representative subset of the target set. This enables us to replace the target set with a smaller one, which is necessary when the set is too big or cannot be calculated. Our method uses LP problems, eliminating the need for MILP techniques and resulting in shorter execution times.

For our algorithm tests, we utilized three models: *iML1515* and version 1.19 of *Human1* to assess the effectiveness of our algorithm in computing essential and synthetic lethality genes in medium and large-scale networks. Lastly, we used the *iJO1366* model to examine potential genetic interventions for ethanol and biomass growth coupling under anaerobic restriction. We have demonstrated that our method meets the requirements and outperforms alternative techniques in the case studies.

The technique discussed in our paper has the potential to improve the use of gMCS in various fields, including medicine. Our approach can target essential genes or multiple combinations of genes (synthetic lethals), which can aid in curing certain illnesses. Furthermore, this technique can also be used in biotechnology to enhance the creation of new byproducts.

## Supplementary Material

vbaf127_Supplementary_Data

## Data Availability

The software, models, and all computed gMCSs can be accessed by visiting https://github.com/biogacop/fastMethod
