# Supplementary material for: A fast method for extracting essential and synthetic lethality genes in GEM models
Source: Bioinform Adv. 2025 Jun 6;5(1):vbaf127. doi: 10.1093/bioadv/vbaf127 (PMC12240467; doi:10.1093/bioadv/vbaf127)
Supplement: vbaf127_Supplementary_Data [file vbaf127_supplementary_data.pdf]

# Supplementary Material

## 1 Time comparison between gMCSPy and Fast Method

Table 1 shows the average time differences between the two methods for computing all gMCSs of length $\leq 4$ across various models, based on five running times. We have used the testbed described in the results section with 64 cores.

We have emphasized instances where the Fast Method has a shorter running time.

The first 25 models are available from BIGG Models `https://bigg.ucsd.edu/` whilst Human1 v1.19.0 can be obtained from `https://github.com/SysBioChalmers/Human-GEM`

| Model | gMCSs | FastMethod | gMCSPy |
|---|---|---|---|
| e coli core | 2486 | 8.56 | 2.07 |
| ic_1306 | 589 | 159.05 | 1802.94 |
| iE2384C_1286 | 696 | 161.28 | 1155.20 |
| iEC1364_W | 727 | 180.18 | 1905.74 |
| iECB_1328 | 723 | 217.51 | 1602.61 |
| iECDH1ME8569_1439 | 696 | 224.85 | 900.85 |
| iECIAI1_1343 | 756 | 265.23 | 819.94 |
| iECO26_1355 | 770 | 204.42 | 1997.71 |
| iECS88_1305 | 774 | 246.47 | 1335.29 |
| iECUMN_1333 | 705 | 249.50 | 768.85 |
| iG2583_1286 | 1209 | 339.27 | 1328.28 |
| iIS312_Trypomastigote | 108 | 37.16 | 1.09 |
| iJN746 | 556 | 492.82 | 421.65 |
| iLJ478 | 421 | 45.45 | 7.01 |
| iNF517 | 481 | 58.98 | 16.45 |
| iS_1188 | 1130 | 405.63 | 814.68 |
| iSF_1195 | 1028 | 520.27 | 741.67 |
| iUMN146_1321 | 732 | 259.72 | 1855.98 |
| iYL1228 | 1492 | 312.39 | 520.67 |
| STM_v1_0 | 809 | 553.96 | 732.79 |
| iAF692 | 7570 | 41.26 | 17.15 |
| iAM_Pk459 | 1604 | 1351.76 | 35.17 |
| iECB_1328 | 723 | 260.47 | 912.62 |
| iML1515 | 889 | 173,99 | 1612.13 |
| iLB1027_lipid | 3125 | 1814.72 | 4671.16 |
| Human-GEM (v1.19) | 145 | 339.71 | 19.05 |
| Human-GEM (v1.19) under Ham's medium | 2549 | 843.09 | 1100.63 |

Table 1: Time in seconds for obtaining synthetic lethalities of length $\leq 4$ for 27 different models

## 2 Comparison of execution times for gMCS computations using gMCSPy and the Fast Method, along with the solvers Gurobi and CPLEX.

Figures 1, 2, 3, 4 and 1 present boxplots of execution times from ten runs for computing all gMCSs of length $\leq 4$ for the models iAF692, iECUMN_1333, iLJ478, iYL1228 and Human1_v1.19 using both Fast Method and gMCSPy, alongside the Gurobi and CPLEX solvers.

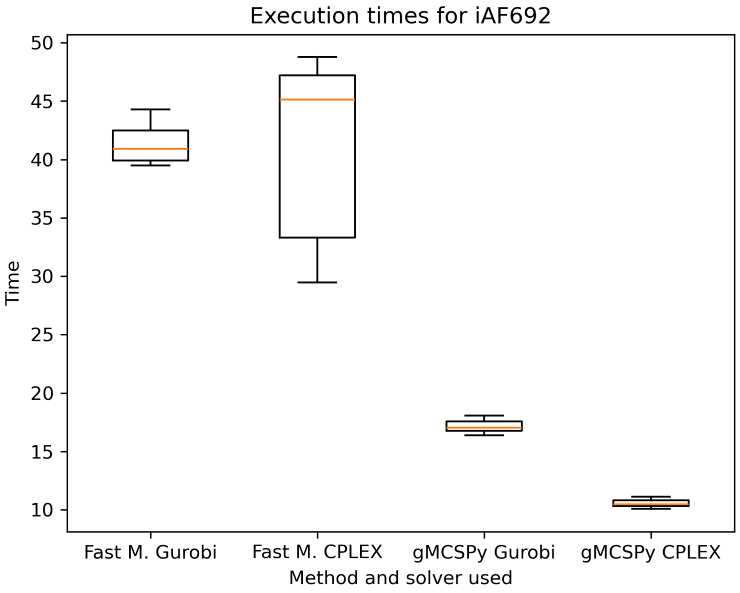


Figure 1: Boxplots showing the variation in the execution times for calculating all gMCSs of lengths $\leq 4$ in iAF1692 using both Fast Method and gMCSPy, alongside the Gurobi and CPLEX solvers

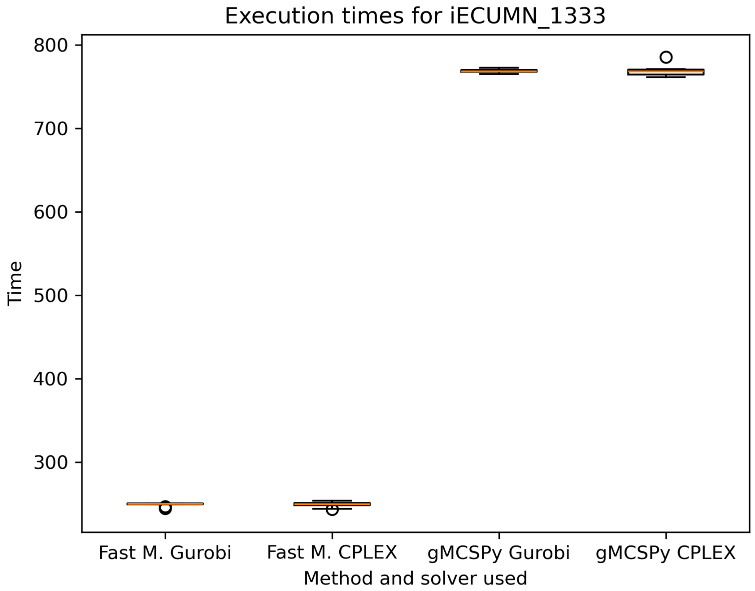


Figure 2: Boxplots showing the variation in the execution times for calculating all gMCSs of lengths $\leq 4$ in iECUMN_1333 using both Fast Method and gMCSPy, alongside the Gurobi and CPLEX solvers

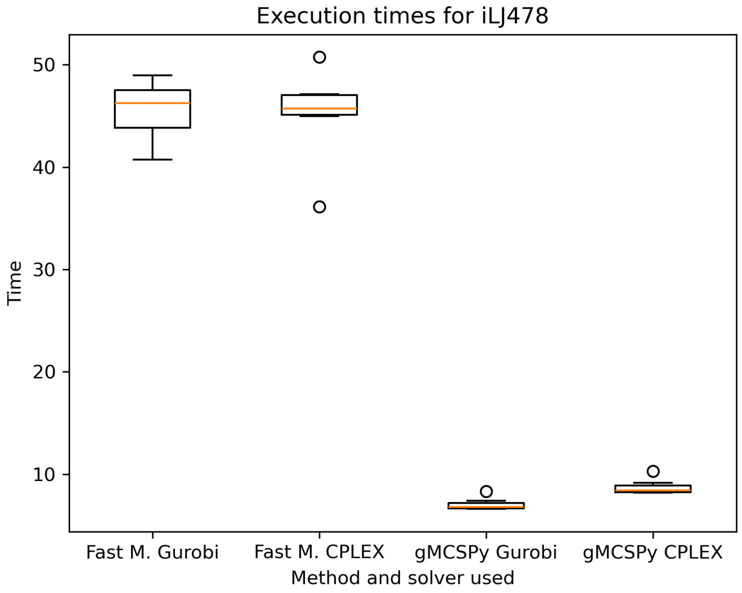


Figure 3: Boxplots showing the variation in the execution times for calculating all gMCSs of lengths $\leq 4$ in iLJ478 using both Fast Method and gMCSPy, alongside the Gurobi and CPLEX solvers

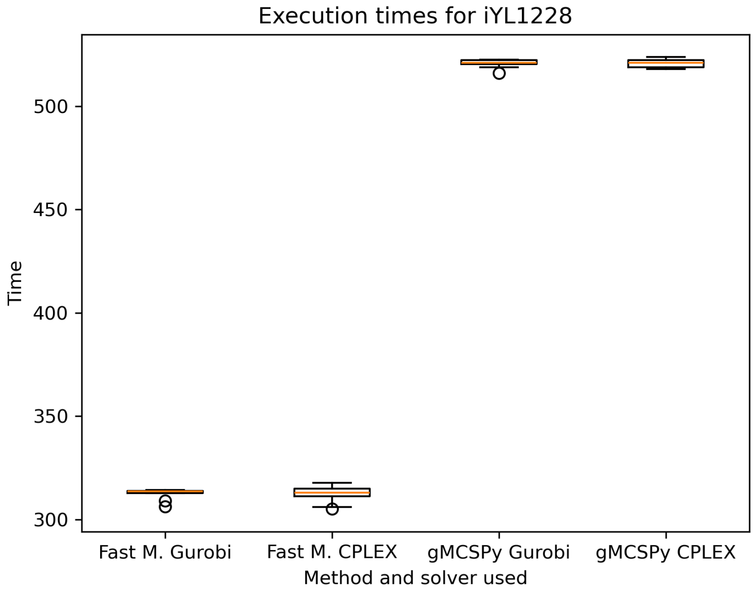


Figure 4: Boxplots showing the variation in the execution times for calculating all gMCSs of lengths $\leq 4$ in iYL1228 using both Fast Method and gMCSPy, alongside the Gurobi and CPLEX solvers

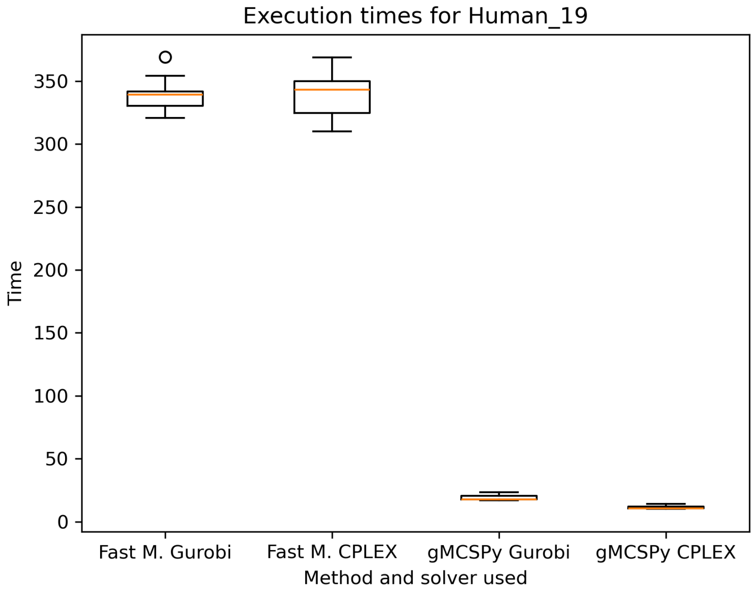


Figure 5: Boxplots showing the variation in the execution times for calculating all gMCSs of lengths $\leq 4$ in Human1_v1.19 using both Fast Method and gMCSPy, alongside the Gurobi and CPLEX solvers
